# Association of Daily Step Patterns With Mortality in US Adults

**DOI:** 10.1001/jamanetworkopen.2023.5174

**Published:** 2023-03-28

**Authors:** Kosuke Inoue, Yusuke Tsugawa, Elizabeth Rose Mayeda, Beate Ritz

**Affiliations:** 1 Department of Social Epidemiology, Graduate School of Medicine, Kyoto University, Kyoto, Japan; 2 Division of General Internal Medicine and Health Services Research, David Geffen School of Medicine at UCLA, Los Angeles, California; 3 Department of Health Policy and Management, Fielding School of Public Health, University of California, Los Angeles; 4 Department of Epidemiology, Fielding School of Public Health, University of California, Los Angeles; 5 Department of Environmental Health Sciences, Fielding School of Public Health, University of California, Los Angeles; 6 Department of Neurology, David Geffen School of Medicine at UCLA, Los Angeles, California

## Abstract

**Importance:**

Previous studies have shown that individuals who regularly walk, particularly 8000 daily steps or more, experience lower mortality. However, little is known about the health benefits of walking intensively only a few days a week.

**Objective:**

To evaluate the dose-response association between the number of days an individual takes 8000 steps or more and mortality among US adults.

**Design, Setting, and Participants:**

This cohort study evaluated a representative sample of participants aged 20 years or older in the National Health and Nutrition Examination Surveys 2005-2006 who wore an accelerometer for 1 week and their mortality data through December 31, 2019. Data were analyzed from April 1, 2022, to January 31, 2023.

**Exposures:**

Participants were grouped by the number of days per week they took 8000 steps or more (0 days, 1-2 days, and 3-7 days).

**Main Outcomes and Measures:**

Multivariable ordinary least squares regression models were used to estimate adjusted risk differences (aRDs) for all-cause and cardiovascular mortality during the 10-year follow-up, adjusting for potential confounders (eg, age, sex, race and ethnicity, insurance status, marital status, smoking, comorbidities, and average daily step counts).

**Results:**

Among 3101 participants (mean [SD] age, 50.5 [18.4] years; 1583 [51.0%] women and 1518 [49.0%] men; 666 [21.5%] Black, 734 [23.7%] Hispanic, 1579 [50.9%] White, and 122 [3.9%] other race and ethnicity), 632 (20.4%) did not take 8000 steps or more any day of the week, 532 (17.2%) took 8000 steps or more 1 to 2 days per week, and 1937 (62.5%) took 8000 steps or more 3 to 7 days per week. Over the 10-year follow-up, all-cause and cardiovascular deaths occurred in 439 (14.2%) and 148 (5.3%) participants, respectively. Compared with participants who walked 8000 steps or more 0 days per week, all-cause mortality risk was lower among those who took 8000 steps or more 1 to 2 days per week (aRD, −14.9%; 95% CI −18.8% to −10.9%) and 3 to 7 days per week (aRD, −16.5%; 95% CI, −20.4% to −12.5%). The dose-response association for both all-cause and cardiovascular mortality risk was curvilinear; the protective association plateaued at 3 days per week. Different thresholds for the number of daily steps between 6000 and 10 000 yielded similar results.

**Conclusions and Relevance:**

In this cohort study of US adults, the number of days per week taking 8000 steps or more was associated with a lower risk of all-cause and cardiovascular mortality in a curvilinear fashion. These findings suggest that individuals may receive substantial health benefits by walking just a couple days a week.

## Key Points

**Question:**

What is the association between the number of days taking a sufficient number of steps throughout the week and mortality among US adults?

**Findings:**

In this cohort study of 3101 adult participants, a curvilinear dose-response association was found between the number of days taking 8000 steps or more throughout the week and a lower risk of all-cause and cardiovascular mortality at 10 years. Participants who only took 8000 steps or more 1 or 2 days during the week also showed substantially lower all-cause and cardiovascular mortality risk.

**Meaning:**

The study’s findings suggest that for adults who face difficulties in exercising regularly, achieving the recommended daily steps only a couple days a week may have meaningful health benefits.

## Teaser

This cohort study uses National Health and Nutrition Examination Survey data to evaluate the association between number of days per week of recommended daily steps and mortality in adults.

## Introduction

Physical inactivity is one of the major public health issues worldwide, contributing to an estimated 3.2 million deaths and imposing $54 billion in direct health care costs annually.^1,2^ Several studies have used the number of daily steps as a simple and valid measure of physical activity and have investigated the association of daily steps with health outcomes, including cardiovascular disease and dementia.^3,4,5,6^ Recently, a meta-analysis suggested that more daily steps are associated with a steady decline in mortality risk up to approximately 8000 daily steps, at which point mortality risk appeared to plateau.^7^ However, according to a study that relied on smartphone accelerometers, the average number of daily steps in US residents was only 4800,^8^ far below the number that has been shown to have substantial benefits, including a reduction in mortality risk. Given the challenges for some people to take a larger number of steps every day, it is important to further elucidate the minimum days throughout a week one should walk the recommended daily steps to incur health benefits.

In modern society, lack of time is one of the major barriers to exercise.^9^ Some individuals choose to concentrate their physical activity into 1 or 2 sessions per week, eg, “weekend warriors” who exercise mainly during the weekend.^10^ Previous studies have shown lower all-cause and cardiovascular mortality risks among weekend warriors compared with people consider to be physically inactive.^10,11,12^ Moreover, the estimated health benefit for weekend warriors was similar to that for people with regular physical activity patterns, indicating that concentration of physical activity into 1 or 2 sessions per week may be sufficient to reduce mortality risks. Although a few studies used objective measurements recorded by wearable devices (eg, accelerometers),^13^ most studies derived data on physical activity levels from the time and intensity of self-reported activities, which raises concerns about the accuracy of data due to social desirability bias (ie, participants may overreport physical activity because it is deemed as socially desirable). Furthermore, to our knowledge, evidence is lacking about the number of days per week one needs to take a certain number of steps (a simpler and more convenient measurement than calculating time and intensity of physical activity^14,15^) to prevent long-term adverse health outcomes.

To address this knowledge gap, we used a nationally representative sample of US adults to investigate the dose-response association of the number of days taking 8000 steps or more (equal to walking approximately 4 miles) throughout a week based on an accelerometer with all-cause and cardiovascular mortality over 10 years. Refining our understanding of the association between daily step patterns and mortality risk based on an objective and convenient measurement may provide additional information to clinicians and decision makers, encouraging them to recommend a minimum number of days in a week that individuals need to achieve a sufficient number of steps to reduce mortality risk.

## Methods

### Study Design and Setting

We used data from the 2005 and 2006 National Health and Nutrition Examination Survey (NHANES) (eMethods 1 in Supplement 1) with linkage to the National Death Index until 2019.^16^ Among 4372 participants aged 20 years or older for whom daily step counts based on accelerometer data were available, 3120 had information on 4 or more valid days of wearing an accelerometer (described below). We excluded participants who lacked data on insurance status (n = 3), body mass index (BMI) as measured by weight in kilograms divided by height in meters squared (n = 15), and follow-up data on mortality at 10 years (n = 1). The research ethics review board of the National Center for Health Statistics approved the study protocol, and all participants gave written informed consent.^17^ The final cohort for analysis included 3101 participants. This study followed the Strengthening the Reporting of Observational Studies in Epidemiology (STROBE) reporting guideline.^18^

### Exposure Ascertainment

In 2005-2006, NHANES participants were asked to wear an accelerometer (ActiGraph model 7164; ActiGraph, LLC) at the waist during waking hours for 7 consecutive days.^19^ Step counts were recorded over 60-second intervals each day.^19^ The validity of step counts collected with the ActiGraph model 7164 has been confirmed in a previous study.^20^ Nonwear time was defined as 60 consecutive minutes or more of 0 counts per minute.^19^ To evaluate the daily step patterns over 1 week, we included individuals with at least 4 days of 10 hours or more of wear time during which step counts were recorded by the accelerometer.^19^ We categorized participants into 3 groups according to the number of days per week they took 8000 steps or more: 0 days, 1 to 2 days, and 3 to 7 days (similar to the definition of weekend warriors for physical activity levels^10,11,12^). Average daily step counts were computed by summing daily steps across the period and dividing them by the number of days with sufficient hours of measurement for each participant.

### Mortality Ascertainment

The primary outcome was all-cause mortality at 10 years of follow-up using death certification information based on the National Death Index through record matching by Social Security number, name, date of birth, race and ethnicity, sex, state of birth, and state of residence.^16^ The secondary outcome was cardiovascular mortality at 10 years of follow-up defined by underlying causes of death based on *International Classification of Diseases, Tenth Revision* codes I00 to I09, I11, I13, I20 to I51, and I60 to I69. Details on covariates are described in eMethods 2 in Supplement 1.

### Statistical Analysis

Analyses were conducted between April 1, 2022, and January 31, 2023. We used multivariable ordinary least squares (OLS) models with Huber-White robust SEs to estimate adjusted risk differences (aRDs) and 95% CIs for all-cause and cardiovascular mortality after 10 years for each activity group defined by the number of days they took 8000 steps or more during the week (1-2 days and 3-7 days; reference group, 0 days). Model 1 included adjustment for age; sex; self-reported race and ethnicity; insurance status; marital status; smoking; BMI; estimated glomerular filtration rate (eGFR); statin use; and history of diabetes, hypertension, cardiovascular disease, cancer, and emphysema. In model 2 (our primary model), we further adjusted for average daily step count. Step intensity was not included in the model, given that a previous study using this cohort showed a null association between step intensity and mortality.^3^

To further evaluate a possible dose-response association between the number of days per week participants took 8000 steps or more and mortality, we used restricted cubic spline functions with 3 knots at the 10th, 50th, and 90th percentiles.^21^ In addition, to assess patterns of association by age and sex, we included additive interaction terms between exposure and age (<65 years vs ≥65 years) or sex (men vs women) in our final OLS regression model. For these analyses, we calculated the adjusted probability for all-cause mortality in each group based on the OLS regression model using the margins command in Stata; that is, we estimated the probability in each subgroup by fixing individual characteristics at each level of the categories and averaged over our national sample (known as the marginal standardization form of predictive margins).^22^

We also conducted several sensitivity analyses (eMethods 3 in Supplement 1). In all analyses, NHANES survey weights were applied to account for unequal probabilities of selecting participants and nonresponse of individuals eligible and approached.^23^ Statistical analyses were conducted using R, version 4.0.2 (R Foundation for Statistical Computing) and Stata, version 17 (StataCorp LLC) software. Statistical code examples are described in eMethods 4 in Supplement 1. All hypothesis tests were 2-sided, and *P* < .05 was considered statistically significant.

## Results

The analytic sample included 3101 participants (mean [SD] age, 50.5 [18.3] years; 1583 [51.0%] women and 1518 [49.0%] men; 666 [21.5%] Black, 734 [23.7%] Hispanic, 1579 [50.9%] White, and 122 [3.9%] other race and ethnicity). The median (IQR) of the average daily step counts was 8793 (6238-11 439) steps. Among the participants, 632 (20.4%) took 8000 steps or more 0 days per week, 532 (17.2%) took 8000 steps or more 1 to 2 days per week, and 1937 (62.5%) took 8000 or more steps 3 to 7 days per week. Participants with a higher number of days taking 8000 steps or more throughout the week were more likely to be younger, men, Hispanic, insured, married, and never smokers and less likely to have obesity, use statins, have comorbidities (ie, decreased eGFR, diabetes, hypertension, cardiovascular disease, cancer, emphysema), have mobility limitations, or rate their health as fair or poor compared with those with a lower number of days per week taking 8000 steps or more (Table 1).

**Table 1.** Demographic Characteristics According to the Number of Days Taking 8000 Steps or More Throughout the Week

Variable	Days per week taking ≥8000 steps, No. (%) of participants		
	0	1-2	3-7
No. of participants	632 (20.4)	532 (17.2)	1937 (62.5)
Age, mean (SD), y	63.7 (19.3)	51.5 (19.0)	46.0 (15.5)
Sex			
Men	269 (42.6)	219 (41.2)	1030 (53.2)
Women	363 (57.4)	313 (58.8)	907 (46.8)
Race and ethnicity			
Black	158 (25.0)	130 (24.4)	378 (19.5)
Hispanic	92 (14.6)	111 (20.9)	531 (27.4)
White	361 (57.1)	269 (50.6)	949 (49.0)
Other^a^	21 (3.3)	22 (4.1)	79 (4.1)
Insurance status			
Uninsured	238 (37.7)	143 (26.9)	292 (15.1)
Private insurance	72 (11.4)	98 (18.4)	469 (24.2)
Public insurance	322 (50.9)	291 (54.7)	1176 (60.7)
Marital status			
Married	300 (47.5)	315 (59.2)	1197 (61.8)
Not married	332 (52.5)	217 (40.8)	740 (38.2)
Smoking status			
Current	109 (17.3)	96 (18.1)	388 (20.0)
Former	223 (35.3)	156 (29.3)	488 (25.2)
Never	300 (47.5)	280 (52.6)	1061 (54.8)
BMI, mean (SD)	30.3 (8.6)	29.5 (6.6)	28.0 (5.6)
eGFR, mean (SD), mL/min/1.73 m^2^	77.0 (25.9)	90.4 (24.0)	95.6 (19.8)
Statin use	360 (20.7)	179 (13.0)	114 (9.4)
History			
Diabetes	128 (20.3)	61 (11.5)	146 (7.5)
Hypertension	354 (56.0)	214 (40.2)	499 (25.8)
Cardiovascular disease	188 (29.8)	63 (11.8)	99 (5.1)
Cancer	116 (18.4)	62 (11.7)	112 (5.8)
Emphysema	35 (5.5)	17 (3.2)	15 (0.8)
Mobility limitation^b^	319 (50.5)	108 (20.3)	132 (6.8)
Self-rated health			
Excellent	34 (5.4)	49 (9.2)	202 (10.4)
Very good	118 (18.7)	153 (28.8)	611 (31.5)
Good	222 (35.1)	195 (36.6)	719 (37.1)
Fair or poor	213 (33.7)	109 (20.5)	298 (15.4)
Missing	45 (7.1)	26 (4.9)	107 (5.5)
Poverty income ratio, mean (SD), %	2.3 (1.5)	2.8 (1.6)	2.9 (1.6)
Missing	43 (6.8)	25 (4.7)	61 (3.2)
Daily step counts throughout the week, median (IQR)	4345 (3229-5262)	6657 (5963-7330)	10 746 (9082-13 193)

Abbreviations: BMI, body mass index as measured by weight in kilograms divided by height in meters squared; eGFR, estimated glomerular filtration rate.

^a^ Other race and ethnicity includes American Indian or Alaska Native, Asian, Native Hawaiian or Pacific Islander, multiple races or ethnicities, or unknown.

^b^ Mobility limitations were defined as self-report of difficulty walking 0.25 miles without special equipment or climbing 10 stairs. Participants who did not report these difficulties were considered to have no mobility limitations.

### Number of Days Taking Recommended Steps and All-Cause and Cardiovascular Mortality

**Figure 1.**
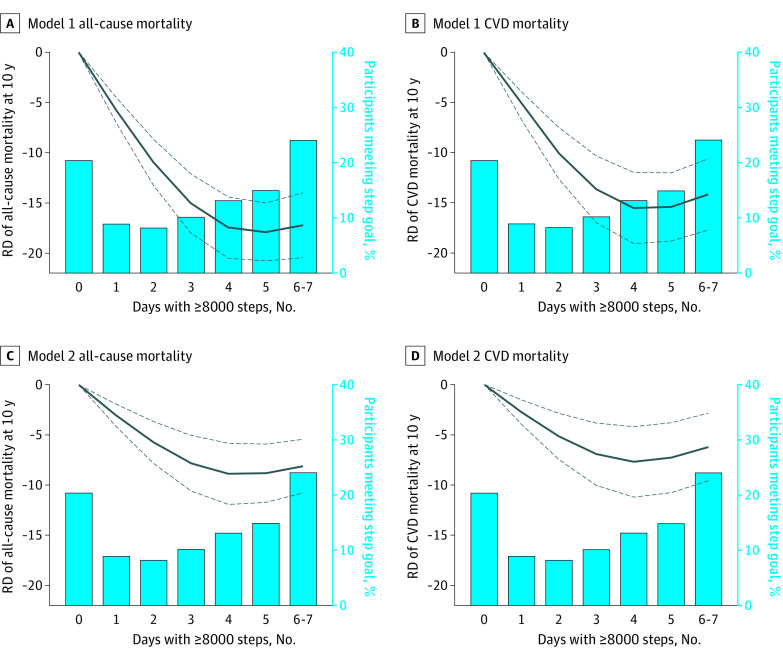
Number of Days Taking 8000 Steps or More Throughout the Week and 10-Year Risk of All-Cause and Cardiovascular Mortality With and Without Adjusting for Average Daily Step Counts Solid lines indicate the point estimate; dash lines, 95% CIs. Model 1 included age; sex; race and ethnicity; insurance status; marital status; smoking; body mass index; estimated glomerular filtration rate; statin use; and history of diabetes, hypertension, cardiovascular disease (CVD), cancer, and emphysema. Model 2 included average daily step counts in addition to the covariates in model 1. RD indicates risk difference.

Over the 10 years of follow-up, 439 deaths (14.2%) from all causes and 148 (5.3%) from cardiovascular disease were identified. After adjusting for potential confounders, compared with participants who took 8000 steps or more 0 days per week, 10-year all-cause mortality risk was 14.9% lower among those who took 8000 steps or more 1 to 2 days per week (aRD, −14.9%; 95% CI, −18.8% to −10.9%) and 16.5% lower among those who took 8000 steps or more 3 to 7 days per week (aRD, −16.5%; 95% CI, −20.4% to −12.5%) on the absolute scale (Table 2; eFigure 1 in Supplement 1). Likewise, 10-year cardiovascular mortality risk was 8.1% lower among those who took 8000 steps or more 1 to 2 days per week (aRD, −8.1%; 95% CI, −11.8% to −4.4%) and 8.4% lower among those who took 8000 steps or more 3 to 7 days per week (aRD, −8.4%; 95% CI, −12.5% to −4.4%) compared with 0 days per week. The restricted cubic spline model showed a curvilinear association between the number of days per week taking 8000 steps or more and the risk of all-cause and cardiovascular mortality; the risk of mortality at 10 years rapidly decreased as the number of days per week taking 8000 steps or more increased and plateaued at 3 to 4 days (Figure 1). This pattern was consistently observed even after adjustment for average daily step counts.

**Table 2.** Adjusted Risk Difference (aRD) for Associations of Number of Days Taking 8000 Steps or More Throughout the Week With All-Cause and Cardiovascular Mortality at 10 Years With and Without Adjusting for Average Daily Step Counts^a^

	Event, No./Total (%)	aRD, % (95% CI)		
		Age and sex	Model 1	Model 2
**All-cause mortality at 10 y**				
Daily steps ≥8000, d/wk				
0	257/632 (40.7)	[Reference]	[Reference]	[Reference]
1-2	75/532 (14.1)	−18.7 (−23.2 to −14.4)	−15.9 (−20.0 to −11.8)	−14.9 (−18.8 to −10.9)
3-7	107/1937 (5.5)	−23.0 (−26.8 to −19.3)	−19.4 (−23.0 to −15.8)	−16.5 (−20.4 to −12.5)
**Cardiovascular mortality at 10 y**				
Daily steps ≥8000, d/wk				
0	87/462 (18.8)	[Reference]	[Reference]	[Reference]
1-2	26/483 (5.4)	−9.6 (−12.9 to −6.3)	−8.5 (−12.2 to −4.9)	−8.1 (−11.8 to −4.4)
3-7	35/1865 (1.9)	−11.1 (−14.4 to −7.9)	−9.8 (−13.4 to −6.2)	−8.4 (−12.5 to −4.4)

^a^ Model 1 included age; sex; race and ethnicity; insurance status; marital status; smoking; body mass index; estimated glomerular filtration rate; statin use; and history of diabetes, hypertension, cardiovascular disease, cancer, and emphysema. Model 2 included average daily step counts in addition to the covariates in model 1. The results of sensitivity analysis additionally adjusting for income, mobility limitation, and self-rated health status are shown in eTable 3 in Supplement 1.

### Stratified Analyses by Age and Sex

**Figure 2.**
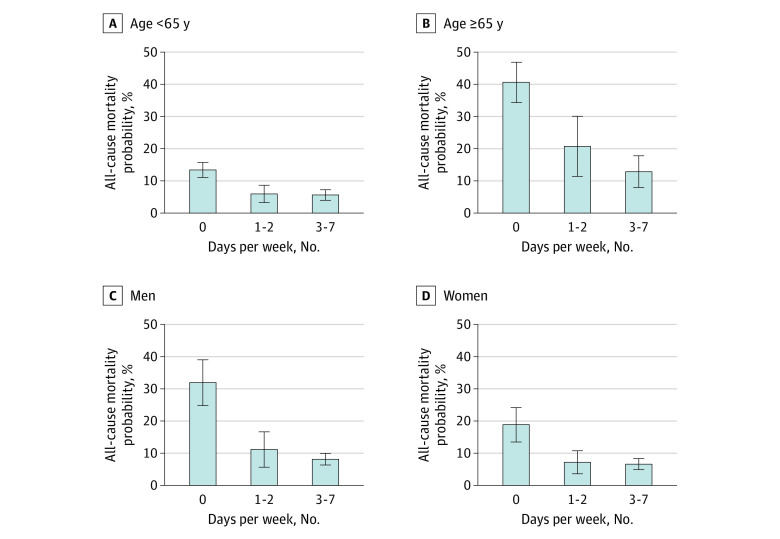
Subgroup Analyses for the Association Between the Number of Days Taking 8000 Steps or More Throughout the Week and 10-Year All-Cause Mortality Risk by Age and Sex Based on the regression models, adjusted probability for all-cause mortality in each exposure status was estimated, fixing individual characteristics at each level of the categories and averaging over the national sample. The model adjusted for age; sex; race and ethnicity; insurance status; marital status; smoking; body mass index; estimated glomerular filtration rate; statin use; history of diabetes, hypertension, cardiovascular disease, cancer, and emphysema; and average daily step counts.

In stratified analyses, both younger and older participants experienced lower 10-year all-cause mortality risk when they took 8000 steps or more 1 to 2 days per week (aged <65 years: aRD, −7.4% [95% CI, −11.2% to −3.7%]; aged ≥65 years: aRD, −19.9% [95% CI, −30.8% to −8.9%]; *P* = .04 for interaction) or 3 to 7 days per week (aged <65 years: aRD, −7.8% [95% CI, −10.3% to −5.2%]; aged ≥65 years: aRD, −27.7% [95% CI, −36.5% to −19.0%]; *P* < .001 for interaction) vs 0 days per week (Figure 2; eTable 1 in Supplement 1). We also found lower 10-year all-cause mortality risk regardless of sex when participants took 8000 steps or more daily 1 to 2 days per week (men: aRD, −20.8% [95% CI, −28.6% to −13.0%]; women: aRD, −11.6% [95% CI, −16.5% to −6.7%]; *P* = .07 for interaction) or 3 to 7 days per week (men: aRD, −23.8% [95% CI, −30.4% to −17.2%]; women: −12.2% [−18.3% to −6.1%]; *P* = .03 for interaction) vs 0 days per week (Figure 2; eTable 2 in Supplement 1).

### Sensitivity Analysis Using Different Thresholds of Daily Steps

We found similar patterns when we used different thresholds of recommended daily steps (eFigure 2 in Supplement 1). For example, the adjusted 10-year all-cause mortality risk was 8.1% among participants who took 10 000 steps or more 1 to 2 days per week and 7.3% and 16.7% among those who took 10 000 steps or more 3 to 7 days per week and 0 days per week, respectively. Similarly, the adjusted 10-year cardiovascular mortality risk was 2.4% among participants who took 10 000 steps or more 1 to 2 days per week and 2.3% and 7.0% among those who took 10 000 steps or more 3 to 7 days per week and 0 days per week, respectively.

### Other Sensitivity Analyses

E-values on the relative risk scale for all-cause mortality were 2.50 for 1 to 2 days per week and 2.66 for 3 to 7 days per week vs 0 days per week, indicating that the effect size for an unmeasured confounder to be able to explain the observed association between the daily step patterns and mortality would need to be quite large (ie, risk ratios of >2.50 conditional on measured covariates included in the regression model when no association between daily steps and mortality exists). Likewise, E-values on the relative risk scale for cardiovascular mortality were 2.34 for 1 to 2 days per week and 2.40 for 3 to 7 days per week vs 0 days per week. In the inverse probability weighting approach that included all covariates in model 1, we estimated RDs closer to the null for all-cause mortality (aRD, −7.2% [95% CI, −11.4% to −3.0%] and −10.6% [95% CI, −14.5% to −6.6%] for 1-2 days per week and 3-7 days per week vs 0 days per week, respectively) and cardiovascular mortality (aRD, −2.0% [95% CI, −4.6% to 0.6%] and −4.0% [95% CI, −6.2% to −1.7%] for 1-2 days per week and 3-7 days per week vs 0 days/week, respectively). The estimated RDs were also closer to null when we additionally adjusted for poverty income ratio, mobility limitations, and self-rated health (eTable 3 in Supplement 1). Results did not qualitatively change when we restricted analyses to individuals with a minimum of 6 days with valid accelerometer measurements (eTable 4 in Supplement 1) and to those with at least an average daily step count of 2000 or more (eTable 5 in Supplement 1). In modified Poisson regression models, the adjusted relative risks for all-cause mortality were 0.75 (95% CI, 0.59-0.96) and 0.64 (95% CI, 0.42-0.99) for 1 to 2 days per week and 3 to 7 days per week, respectively, vs 0 days per week (eTable 6 in Supplement 1).

## Discussion

In this cohort study using nationally representative data for US adults, we estimated that a higher number of days taking 8000 steps or more throughout the week is associated with a decreased risk of all-cause and cardiovascular mortality at 10 years. Even participants who only took 8000 steps or more 1 or 2 days during the week showed a substantial reduction in all-cause and cardiovascular mortality compared with those who were active more regularly (ie, took ≥8000 steps for 3-7 days per week). The estimated association was also robust to using different thresholds between 6000 and 10 000 steps for the daily step counts. Our study findings suggest that for those individuals who face difficulties in exercising regularly (eg, due to work and/or family obligations), achieving recommended daily steps only a couple days per week can have meaningful health benefits.

Our findings add to the knowledge base from previous studies on the association between frequency of physical activity and long-term health outcomes. In the Harvard Alumni Health Study that enrolled 8421 men, Lee et al^10^ found that the hazard ratios (HRs) for all-cause mortality among weekend warriors and people engaging in regular exercise compared with those with sedentary behavior were 0.85 (95% CI, 0.65-1.11) and 0.64 (95% CI, 0.55-0.73), respectively. In a population-based cohort of 63 591 adults from the Health Survey for England and the Scottish Health Survey, O’Donovan et al^11^ estimated similar-magnitude HRs for all-cause mortality among weekend warriors (HR, 0.70; 95% CI, 0.60-0.82) and regularly active participants (HR, 0.65; 95% CI, 0.58-0.73) compared with inactive participants. A more recent study in 350 978 participants from the US National Health Interview Survey^12^ supports these findings, wherein all-cause and cause-specific mortality risks did not differ between weekend warriors and regularly active participants, even after accounting for the total amount of moderate to vigorous physical activity. While all these studies defined physical activity based on self-reported duration and intensity of the activity, Shiroma et al^13^ used data from 3438 NHANES participants to show protective associations between accelerometer-assessed higher physical activity and mortality. However, to our knowledge, no studies to date have evaluated whether taking the recommended daily steps only 1 to 2 days per week is sufficient to reduce all-cause and cardiovascular mortality.

In line with the proliferation and popularity of smartphones and wearable devices that count daily steps, monitoring and targeting daily steps has been considered a practical strategy for promoting physical activity in the population by clinicians, patients, and public health professionals.^24^ Indeed, dose-response associations of step count with all-cause mortality and cardiovascular events have been reported in several systematic reviews and meta-analyses.^25,26,27^ Particularly, a recent meta-analysis showed decreasing mortality risks with an increasing number of daily steps until 6000 to 8000 steps are reached for adults older than 60 years and at 8000 to 10 000 steps per day for those younger than 60 years.^7^ Yet, not all people have sufficient time and energy for achieving the recommended daily steps every day of the week in part due to their work and comorbidities, resulting in a lower average number of daily steps taken by US residents.^8^ Given the simplicity and ease of counting daily steps, our findings indicate that the recommended number of steps taken on as few as 1 to 2 days per week may be a feasible option for individuals who are striving to achieve some health benefits through adhering to a recommended daily step count but are unable to accomplish this on a daily basis.

### Strengths and Limitations

Our study has 2 major strengths. First, we used objective measurements of daily steps throughout a week based on accelerometer data, which allowed us to avoid potential bias from exposure misclassification due to faulty self-report. Second, by leveraging the fact that almost all participants (>99.9%) had follow-up data from national death records, we calculated an aRD (absolute scale) for all-cause and cardiovascular mortality after 10 years. Although the previous literature on this topic generally presented HRs, such calculation may obscure the reduction in the mortality risk associated with exposure.^28,29^ In this context, our findings reveal new quantitative evidence for a potential reduction in all-cause and cardiovascular mortality by approximately 15 and 8 percentage points, respectively, when taking 8000 steps or more for at least 1 to 2 days per week.

This study also has several limitations. First, because daily steps were only measured for 1 week at baseline, we did not have information on how changes in physical activity contribute to risk of mortality. Second, given previous work showing that the ActiGraph accelerometer missed 20% of the daily steps when adults with obesity walked at moderate speed,^30^ we cannot rule out the possibility of measurement error of exposure. Moreover, as a result of knowledge that they are wearing the device, participants might have a tendency to be more active, a phenomenon known as the Hawthorne effect.^31,32^ Such reactivity might cause misclassification such that some people who are typically physically inactive were categorized as physically active (based on a reaction to wearing the accelerometer) particularly because NHANES measured their daily step pattern for only 1 week. If only healthy people who are physically less active are able to increase their physical activity levels during the measurement period, this type of misclassification might introduce bias away from the null. Third, covariates such as socioeconomic status and medical history might have been mismeasured due to their self-reported nature, as they were obtained via survey. Fourth, although we included an extensive set of covariates and conducted several sensitivity analyses, our findings still have residual confounding bias (ie, poor health could influence daily step counts and mortality risk). Fifth, our findings might suffer from selection bias due to restricting study samples to those who wore an accelerometer for 1 week during the survey. Sixth, because of the limited number of events, we did not assess cause-specific mortality other than cardiovascular deaths (eg, cancer deaths). Further longitudinal studies with larger sample sizes and time-varying measures of physical activity or step counts would be warranted to overcome this limitation.

## Conclusions

Among US adults, we estimated a curvilinear dose-response association between the number of days taking 8000 steps or more throughout the week and decreased all-cause and cardiovascular mortality risks. The protective association plateaued when individuals took sufficient daily steps for 3 days or more. Although our findings might suffer from residual confounding that should be addressed in future research, they suggest that people may receive substantial health benefits even if a sufficient number of steps are taken on only a couple days of the week.

## Notes

Supplement 1. **eMethods 1.** Details of Study Cohort: The National Health and Nutrition Examination Survey **eMethods 2.** Covariates Ascertainment **eMethods 3.** Details of Sensitivity Analyses **eMethods 4.** Statistical Code Examples **eFigure 1.** Association Between the Number of Days Taking 8000 Steps or More Throughout the Week and 10-Year Mortality Risk **eFigure 2.** Sensitivity Analysis: Adjusted Risk of All-Cause and Cardiovascular Mortality at 10 Years According to Daily Step Patterns Throughout 1 Week Using Different Thresholds of Steps per Day **eTable 1.** Stratified Analyses for the Association Between the Number of Days Taking 8000 Steps or More and the Risks of All-Cause Mortality at 10 Years by Age **eTable 2.** Stratified Analyses for the Association Between the Number of Days Taking 8000 Steps or More and the Risks of All-Cause Mortality at 10 Years by Sex **eTable 3.** Sensitivity Analyses for the Association Between the Number of Days Taking 8000 Steps or More and the Risks of All-Cause and Cardiovascular Mortality at 10 Years Additionally Adjusted for Poverty Income Ratio, Mobility Limitations, and Self-Rated Health Status (n = 2810) **eTable 4.** Sensitivity Analyses for the Association Between the Number of Days Taking 8000 or More Steps and the Risks of All-Cause and Cardiovascular Mortality at 10 Years Among Individuals With a Minimum of 6 Valid Days of Accelerometer Measurements Throughout the Week (n = 2252) **eTable 5.** Sensitivity Analyses for the Association Between the Number of Days Taking 8000 or More Steps and the Risks of All-Cause and Cardiovascular Mortality at 10 Years Among Individuals With at Least an Average Daily Step Count of 2000 or More (n = 3052) **eTable 6.** Sensitivity Analyses for the Association Between the Number of Days Taking 8000 or More Steps and the Risks of All-Cause and Cardiovascular Mortality at 10 Years After Using Modified Poisson Regression Models (on Relative Scale)
eReferences.

Supplement 2.
Data Sharing Statement
