# Error in Figure 1

**DOI:** 10.1001/jamanetworkopen.2023.11413

**Published:** 2023-04-19

In the Original Research titled “Association of Daily Step Patterns With Mortality in US Adults,”^1^ published March 28, 2023, in Figure 1, the panel labels and y-axis labels for panels B and C were reversed; panel B represents model 2 all-cause mortality, and panel C represents model 1 CVD mortality. This article has been corrected.^1^
