# Supplementary material for: Association of Daily Step Patterns With Mortality in US Adults
Source: JAMA Netw Open. 2023 Mar 28;6(3):e235174. doi: 10.1001/jamanetworkopen.2023.5174 (PMC10051082; doi:10.1001/jamanetworkopen.2023.5174)
Supplement: Supplement 1. — eMethods 1. Details of Study Cohort: The National Health and Nutrition Examination Survey eMethods 2. Covariates Ascertainment eMethods 3. Details of Sensitivity Analyses eMethods 4. Statistical Code Examples eFigure 1. Association Between the Number of Days Taking 8000 Steps or More Throughout the Week and 10-Year Mortality Risk eFigure 2. Sensitivity Analysis: Adjusted Risk of All-Cause and Cardiovascular Mortality at 10 Years According to Daily Step Patterns Throughout 1 Week Using Different Thresholds of Steps per Day eTable 1. Stratified Analyses for the Association Between the Number of Days Taking 8000 Steps or More and the Risks of All-Cause Mortality at 10 Years by Age eTable 2. Stratified Analyses for the Association Between the Number of Days Taking 8000 Steps or More and the Risks of All-Cause Mortality at 10 Years by Sex eTable 3. Sensitivity Analyses for the Association Between the Number of Days Taking 8000 Steps or More and the Risks of All-Cause and Cardiovascular Mortality at 10 Years Additionally Adjusted for Poverty Income Ratio, Mobility Limitations, and Self-Rated Health Status (n = 2810) eTable 4. Sensitivity Analyses for the Association Between the Number of Days Taking 8000 or More Steps and the Risks of All-Cause and Cardiovascular Mortality at 10 Years Among Individuals With a Minimum of 6 Valid Days of Accelerometer Measurements Throughout the Week (n = 2252) eTable 5. Sensitivity Analyses for the Association Between the Number of Days Taking 8000 or More Steps and the Risks of All-Cause and Cardiovascular Mortality at 10 Years Among Individuals With at Least an Average Daily Step Count of 2000 or More (n = 3052) eTable 6. Sensitivity Analyses for the Association Between the Number of Days Taking 8000 or More Steps and the Risks of All-Cause and Cardiovascular Mortality at 10 Years After Using Modified Poisson Regression Models (on Relative Scale) eReferences. [file jamanetwopen-e235174-s001.pdf]

Supplemental Online Content

Inoue K, Tsugawa Y, Mayeda ER, Ritz B. Association of daily step patterns with mortality in US adults. *JAMA Netw Open*. 2023;6(3):e235174. doi:10.1001/jamanetworkopen.2023.5174

eMethods 1. Details of Study Cohort: The National Health and Nutrition Examination Survey

eMethods 2. Covariates Ascertainment

eMethods 3. Details of Sensitivity Analyses

eMethods 4. Statistical Code Examples

eFigure 1. Association Between the Number of Days Taking 8000 Steps or More Thoughout the Week and 10-Year Mortality Risk

eFigure 2. Sensitivity Analysis: Adjusted Risk of All-Cause and Cardiovascular Mortality at 10 Years According to Daily Step Patterns Throughout 1 Week Using Different Thresholds of Steps per Day

eTable 1. Stratified Analyses for the Association Between the Number of Days Taking 8000 Steps or More and the Risks of All-Cause Mortality at 10 Years by Age

eTable 2. Stratified Analyses for the Association Between the Number of Days Taking 8000 Steps or More and the Risks of All-Cause Mortality at 10 Years by Sex

eTable 3. Sensitivity Analyses for the Association Between the Number of Days Taking 8000 Steps or More and the Risks of All-Cause and Cardiovascular Mortality at 10 Years Additionally Adjusted for Poverty Income Ratio, Mobility Limitations, and Self-Rated Health Status (n = 2810)

eTable 4. Sensitivity Analyses for the Association Between the Number of Days Taking 8000 or More Steps and the Risks of All-Cause and Cardiovascular Mortality at 10 Years Among Individuals With a Minimum of 6 Valid Days of Accelerometer Measurements Throughout the Week (n = 2252)

eTable 5. Sensitivity Analyses for the Association Between the Number of Days Taking 8000 or More Steps and the Risks of All-Cause and Cardiovascular Mortality at 10 Years Among Individuals With at Least an Average Daily Step Count of 2000 or More (n = 3052)

eTable 6. Sensitivity Analyses for the Association Between the Number of Days Taking 8000 or More Steps and the Risks of All-Cause and Cardiovascular Mortality at 10 Years After Using Modified Poisson Regression Models (on Relative Scale)

eReferences.

This supplemental material has been provided by the authors to give readers additional information about their work.

© 2023 Inoue K et al. *JAMA Network Open.*

eMethods 1. Details of Study Cohort: The National Health and Nutrition Examination Survey

NHANES is a nationwide multi-stage survey of non-institutionalized US adults, conducted by the National Center for Health Statistics (NCHS) of the Centers for Disease Control and Prevention. For NHANES, participants are selected through a multistage sampling design, and data are collected in structured home interviews, physical examinations, and blood and urine sampling. The Research Ethics Review Board of the National Center for Health Statistics approved the study protocol, and all participants gave written informed consent.[1] The present study includes NHANES 2005-2006 participants who participated in physical examinations, which included wearing an accelerometer to measure daily steps. The response rates of the household interview and examinations conducted for the NHANES 2005-2006 were 80.5% and 77.4%, respectively.[2]

© 2023 Inoue K et al. *JAMA Network Open.*

eMethods 2. Covariates Ascertainment

Demographic characteristics included age, gender (men, women), self-reported race/ethnicity (Hispanic, non-Hispanic Black, non-Hispanic White, or others), education status (less than high school, high school or General Education Degree, or more than high school), health insurance status (private, public, none), marital status (single, married), and smoking status (never, former, current). Comorbidities included diabetes mellitus, hypertension, cardiovascular disease, cancer, and emphysema based on self-reported physician diagnoses of these diseases. BMI was calculated from measured weights and heights. An estimated glomerular filtration rate (eGFR, $mL/min/1.73m^2$) was calculated from serum creatinine measurements using the new 2021 Chronic Kidney Disease Epidemiology Collaboration equation.[3] Statin prescription was ascertained from the examination of the containers provided by the participants. Income levels were defined based on family poverty income ratios, which accounted for household size, the number of related children, and inflation in the survey year. Mobility limitations were defined as self-report of difficulty walking 0.25 miles without special equipment or climbing 10 stairs. Self-rated health was assessed using the following question: "Would you say your health in general is excellent, very good, good, fair, or poor?".

© 2023 Inoue K et al. *JAMA Network Open.*

eMethods 3. Details of Sensitivity Analyses

We conducted the following seven sensitivity analyses. First, to assess the sensitivity of our findings to the number of daily steps, we reanalyzed the data using different thresholds of steps per day between 6000 and 10000 steps. Second, we computed the E-value (on the relative risk scale[4]) to quantify the minimum strength of the association of an unmeasured confounder with both the exposure (daily step pattern) and the outcome (mortality), which could explain the observed exposure-outcome association.[5,6] Third, we applied the inverse-probability weighting approach using covariates in Model 1 to consider pseudo-population in which measured covariates were balanced across the exposure groups (estimated using *teffects ipw* in Stata; average daily step counts were not included in this model due to high correlation with the exposure). Fourth, to account for the possibility of confounding by socioeconomic status, we additionally adjusted for poverty income ratio, mobility limitations, and self-rated health in subpopulations for whom such data were available (n=2810). Fifth, to minimize exposure misclassification due to the accuracy of the accelerometer, we restricted analyses to individuals who contributed at least six days of valid data in the measurement week (n=2252). Sixth, as individuals with extremely low or no physical activity might not be comparable to those with at least some activity, we reanalyzed the data after restricting to individuals with at least an average daily step count of ≥2000 (n=3052). Lastly, given the binary outcome of all-cause and cardiovascular mortality at 10 years, we applied modified Poisson regression models to estimate the adjusted odds ratio for each daily step pattern.

© 2023 Inoue K et al. *JAMA Network Open.*

eMethods 4. Statistical Code Examples

****Study sample selection (each individual has 7 days of observations in the original NHANES data)**

```
keep if valid_days>=4 //adults had information on ≥4 valid days of wearing an accelerometer.
"valid_days" were obtained through nhanesaccel package in R
(https://github.com/vandomed/nhanesaccel)
keep if insurance!-. & bmi!=. //complete case analyses for Model 1
```

****Exposure**

```
forvalues i=6000(1000)10000{
capture drop paxdup
gen ach`i'=0 if step<`i'
replace ach`i'=1 if step>=`i'
sort seqn step
by seqn: gen paxdup= cond(_N==1, 0, _n)
by seqn: egen ach`i'total= total(ach`i')
}
capture drop exp
gen exp=1 if ach8000total==0
replace exp=2 if ach8000total>=1 & ach8000total<=2
replace exp=3 if ach8000total>=3 & ach8000total<=7
```

****Outcome**

```
gen mortall_10=0 if permth_exm>120 & permth_exm<. //permth_exm is follow-up months
replace mortall_10=1 if permth_exm<=120 & all_death==1

gen mortcvd_10=0 if permth_exm>120 & permth_exm<.
replace mortcvd_10=1 if permth_exm<=120 & cvd_death==1
```

****Delete all duplicates in the data**

```
bysort seqn: gen dup= cond(_N==1, 0, _n)
keep if dup==1
```

****Main analysis (OLS and modified Poisson)**

```
svyset sdmvpsu [pweight =weight],  strata(sdmvstra) //weight = NHANES survey weight
foreach i in mortall_10 mortcvd_10 {
svy: glm `i' i.exp covariates , family(gaussian) link()
margins i.exp //calculate adjusted probability
svy: glm `i' i.exp covariates , family(poisson) link(log) eform
margins i.exp
}
```

© 2023 Inoue K et al. *JAMA Network Open.*

eFigure 1. Association Between the Number of Days Taking 8000 Steps or More Thoughout the Week and 10-Year Mortality Risk

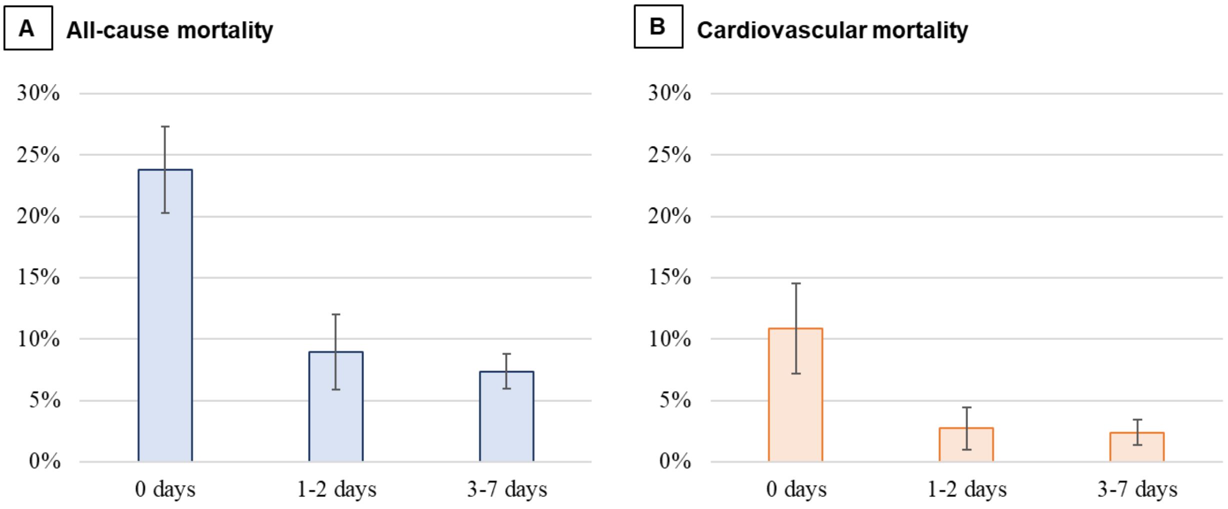

Based on the regression models, we estimated the adjusted probability for (A) all-cause mortality and (B) cardiovascular mortality (y-axis) in each exposure status (x-axis) fixing individual characteristics at each level of the categories and averaged over our national sample. The model adjusted for age, gender, race/ethnicity, insurance status, marital status, smoking, BMI, eGFR, statin use, previous histories of diabetes, hypertension, cardiovascular disease, cancer, and emphysema, and average daily step counts.

© 2023 Inoue K et al. *JAMA Network Open.*

eFigure 2. Sensitivity Analysis: Adjusted Risk of All-Cause and Cardiovascular Mortality at 10 Years According to Daily Step Patterns Throughout 1 Week Using Different Thresholds of Steps per Day

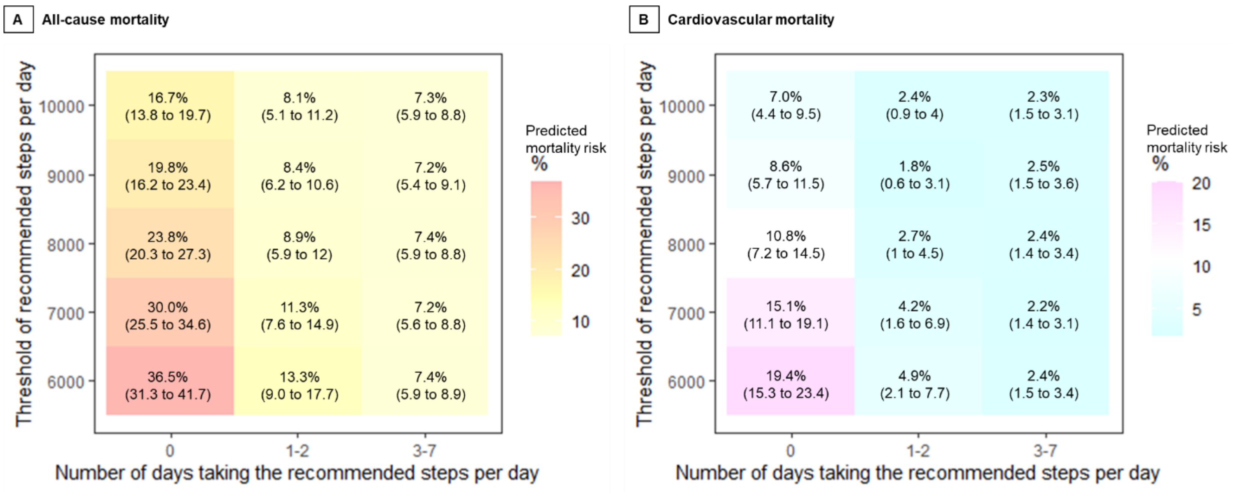

X-axis shows the number of days taking recommended steps per day throughout a week (0 days, 1-2 days, and 3-7 days). Y-axis shows the threshold of recommended steps per day increasing from 6000 to 10000 steps (i.e., each row shows the results for each threshold and these threshold specific results are not mutually exclusive). Based on the regression models, we estimated the adjusted probability for (A) all-cause mortality and (B) cardiovascular mortality by each exposure status fixing individual characteristics at each level of activity and averaged over our national sample. Covariates included in the models were age, gender, race/ethnicity, insurance status, marital status, smoking, BMI, eGFR, statin use, previous histories of diabetes, hypertension, cardiovascular disease, cancer, and emphysema, and average daily step counts. For example, the adjusted risks of all-cause mortality at 10 years were 16.7% among adults who never took ≥10000 steps/day, 8.1% among adults who took ≥10000 steps 1-2 days/week, and 7.3% lower among adults who took ≥10000 steps 3-7 days/week.

© 2023 Inoue K et al. *JAMA Network Open.*

eTable 1. Stratified Analyses for the Association Between the Number of Days Taking 8000 Steps or More and the Risks of All-Cause Mortality at 10 Years by Age

	Event (%)	Adjusted RD (95% CI)	
		**Model 1**	**Model 2**
**<65 years**			
0 day/week with daily steps ≥8000	41/253 (16.2)	ref	ref
1-2 days/week with daily steps ≥8000	20/380 (5.3)	-8.6% (-12.8% to -4.4%)	-7.4% (-11.2% to -3.7%)
3-7 days/week with daily steps ≥8000	47/1658 (2.8)	-11.1% (-14.9% to -7.3%)	-7.8% (-10.3% to -5.2%)
**≥65 years**			
0 day/week with daily steps ≥8000	216/379 (57.0)	ref	ref
1-2 days/week with daily steps ≥8000	55/152 (36.2)	-21.0% (-32.0% to -9.9%)	-19.9% (-30.8% to -8.9%)
3-7 days/week with daily steps ≥8000	60/279 (21.5)	-30.8% (-38.6% to -23.0%)	-27.7% (-36.5% to 19.0%)

Model 1 included age, gender, race/ethnicity, insurance status, marital status, smoking, BMI, eGFR, statin use, and previous histories of diabetes, hypertension, cardiovascular disease, cancer, and emphysema
Model 2 included average daily step counts in addition to the covariates in Model 1.

© 2023 Inoue K et al. *JAMA Network Open.*

eTable 2. Stratified Analyses for the Association Between the Number of Days Taking 8000 Steps or More and the Risks of All-Cause Mortality at 10 Years by Sex

	Event (%)	Adjusted RD (95% CI)	
		**Model 1**	**Model 2**
**Men**			
0 day/week with daily steps ≥8000	140/269 (52.0)	ref	ref
1-2 days/week with daily steps ≥8000	47/219 (21.5)	-21.8% (-30.0% to -13.5%)	-20.8% (-28.6% to -13.0%)
3-7 days/week with daily steps ≥8000	67/1030 (6.5)	-26.6% (-33.5% to -19.7%)	-23.8% (-30.4% to -17.2%)
**Women**			
0 day/week with daily steps ≥8000	117/363 (32.2)	ref	ref
1-2 days/week with daily steps ≥8000	28/313 (9.0)	-12.5% (-17.4% to -7.6%)	-11.6% (-16.5% to -6.7%)
3-7 days/week with daily steps ≥8000	40/907 (4.4)	-14.5% (-20.5% to -8.6%)	-12.2% (-18.3% to -6.1%)

Model 1 included age, gender, race/ethnicity, insurance status, marital status, smoking, BMI, eGFR, statin use, and previous histories of diabetes, hypertension, cardiovascular disease, cancer, and emphysema
Model 2 included average daily step counts in addition to the covariates in Model 1.

© 2023 Inoue K et al. *JAMA Network Open.*

eTable 3. Sensitivity Analyses for the Association Between the Number of Days Taking 8000 Steps or More and the Risks of All-Cause and Cardiovascular Mortality at 10 Years Additionally Adjusted for Poverty Income Ratio, Mobility Limitations, and Self-Rated Health Status (n = 2810)

	**Event (%)**	**Adjusted RD (95% CI)**		
		**Model 1**	**Model 2**	**Model 3**
All-cause mortality at 10 years				
0 day/week with daily steps ≥8000	210/550 (38.2)	ref	ref	ref
1-2 days/week with daily steps ≥8000	65/482 (13.5)	-13.5% (-17.4% to -9.7%)	-12.5% (-16.2% to -8.8%)	-10.1% (-13.6% to -6.6%)
3-7 days/week with daily steps ≥8000	100/1778 (5.6)	-16.8% (-20.0% to -13.6%)	-14.0% (-17.5% to -10.4%)	-10.8% (-14.5% to -7.2%)
Cardiovascular mortality at 10 years				
0 day/week with daily steps ≥8000	71/411 (17.3)	ref	ref	ref
1-2 days/week with daily steps ≥8000	23/440 (5.2)	-7.4% (-10.6% to -4.2%)	-6.9% (-10.2% to -3.7%)	-5.9% (-9.1% to -2.8%)
3-7 days/week with daily steps ≥8000	34/1712 (2.0)	-8.3% (-11.5% to -5.1%)	-6.9% (-10.5% to -3.3%)	-5.5% (-8.8% to -2.2%)

Model 1 included age, gender, race/ethnicity, insurance status, marital status, smoking, BMI, eGFR, statin use, and previous histories of diabetes, hypertension, cardiovascular disease, cancer, and emphysema
Model 2 included average daily step counts in addition to the covariates in Model 1.
Model 3 included poverty income ratio, mobility limitation, and self-rated health status in addition to the covariates in Model 2.

© 2023 Inoue K et al. *JAMA Network Open.*

eTable 4. Sensitivity Analyses for the Association Between the Number of Days Taking 8000 or More Steps and the Risks of All-Cause and Cardiovascular Mortality at 10 Years Among Individuals With a Minimum of 6 Valid Days of Accelerometer Measurements Throughout the Week (n = 2252)

	Event (%)	Adjusted RD (95% CI)	
		**Model 1**	**Model 2**
All-cause mortality at 10 years			
0 day/week with daily steps ≥8000	196/442 (44.3)	ref	ref
1-2 days/week with daily steps ≥8000	58/329 (17.6)	-18.8% (-24.1% to -13.4%)	-17.8 (-22.8% to -12.9%)
3-7 days/week with daily steps ≥8000	87/1481 (5.9)	-22.1% (-26.9% to -17.3%)	-19.1 (-23.9% to -14.2%)
Cardiovascular mortality at 10 years			
0 day/week with daily steps ≥8000	62/308 (20.1)	ref	ref
1-2 days/week with daily steps ≥8000	18/289 (6.2)	-10.4% (-14.6% to -6.1%)	-9.9% (-14.2% to -5.6%)
3-7 days/week with daily steps ≥8000	30/1424 (2.1)	-10.7% (-15.0% to -6.4%)	-9.1% (-14.1% to -4.1%)

Model 1 included age, gender, race/ethnicity, insurance status, marital status, smoking, BMI, eGFR, statin use, and previous histories of diabetes, hypertension, cardiovascular disease, cancer, and emphysema
Model 2 included average daily step counts in addition to the covariates in Model 1.

© 2023 Inoue K et al. *JAMA Network Open.*

eTable 5. Sensitivity Analyses for the Association Between the Number of Days Taking 8000 or More Steps and the Risks of All-Cause and Cardiovascular Mortality at 10 Years Among Individuals With at Least an Average Daily Step Count of 2000 or More (n = 3052)

	**Event (%)**	**Adjusted RD (95% CI)**		
		**Age + Gender**	**Model 1**	**Model 2**
All-cause mortality at 10 years				
0 days/week with daily steps ≥8000	218/583 (37.4%)	ref	ref	ref
1-2 days/week with daily steps ≥8000	75/532 (14.1%)	-16.7% (-21.2% to -12.3%)	-14.2% (-18.4% to -10.0%)	-13.5% (-17.5% to -9.5%)
3-7 days/week with daily steps ≥8000	107/1937 (5.5%)	-21.1% (-24.9% to -17.3%)	-17.8% (-21.4% to -14.2%)	-15.5% (-19.4% to -11.7%)
Cardiovascular mortality at 10 years				
0 days/week with daily steps ≥8000	73/438 (16.7%)	ref	ref	ref
1-2 days/week with daily steps ≥8000	26/483 (5.4%)	-7.9% (-11.6% to -4.2%)	-7.1% (-11.0% to -3.2%)	-6.8% (-10.7% to -2.9%)
3-7 days/week with daily steps ≥8000	35/1865 (1.9%)	-9.5% (-13.1% to -5.8%)	-8.5% (-12.4% to -4.6%)	-7.5% (-11.7% to -3.3%)

Model 1 included age, gender, race/ethnicity, insurance status, marital status, smoking, BMI, eGFR, statin use, and previous histories of diabetes, hypertension, cardiovascular disease, cancer, and emphysema Model 2 included average daily step counts in addition to the covariates in Model 1.

© 2023 Inoue K et al. *JAMA Network Open.*

eTable 6. Sensitivity Analyses for the Association Between the Number of Days Taking 8000 or More Steps and the Risks of All-Cause and Cardiovascular Mortality at 10 Years After Using Modified Poisson Regression Models (on Relative Scale)

	Event (%)	Adjusted relative risk (95% CI)		
		Age + Gender	Model 1	Model 2
All-cause mortality at 10 years				
0 days/week with daily steps ≥8000	257/632 (40.7%)	ref	ref	ref
1-2 days/week with daily steps ≥8000	75/532 (14.1%)	0.58 (0.44 to 0.76)	0.65 (0.51 to 0.82)	0.75 (0.59 to 0.96)
3-7 days/week with daily steps ≥8000	107/1937 (5.5%)	0.35 (0.26 to 0.47)	0.44 (0.31 to 0.61)	0.64 (0.42 to 0.99)
Cardiovascular mortality at 10 years				
0 days/week with daily steps ≥8000	87/462 (18.8%)	ref	ref	ref
1-2 days/week with daily steps ≥8000	26/483 (5.4%)	0.52 (0.36 to 0.73)	0.63 (0.45 to 0.87)	0.81 (0.54 to 1.21)
3-7 days/week with daily steps ≥8000	35/1865 (1.9%)	0.35 (0.21 to 0.57)	0.43 (0.25 to 0.75)	0.83 (0.29 to 2.33)

Model 1 included age, gender, race/ethnicity, insurance status, marital status, smoking, BMI, eGFR, statin use, and previous histories of diabetes, hypertension, cardiovascular disease, cancer, and emphysema
Model 2 included average daily step counts in addition to the covariates in Model 1.

© 2023 Inoue K et al. *JAMA Network Open.*

eReferences

1. National Center for Health Statistics, Centers for Disease Control and Prevention. NCHS research ethics review board (ERB) approval. Published December 1, 2017. Accessed February 18, 2019. www.cdc.gov/nchs/nhanes/irba98.htm

2. National Center for Health Statistics, Centers for Disease Control and Prevention. NHANES response rates and population totals. Accessed February 18, 2019. https://wwwn.cdc.gov/nchs/nhanes/ResponseRates.aspx

3. Inker LA, Eneanya ND, Coresh J, et al. New Creatinine- and Cystatin C–Based Equations to Estimate GFR without Race. *N Engl J Med*. 2021;385(19):1737-1749. doi:10.1056/NEJMoa2102953

4. Ding P, VanderWeele TJ. Sensitivity Analysis Without Assumptions. *Epidemiology*. 2016;27(3):368-377. doi:10.1097/EDE.0000000000000457

5. Haneuse S, VanderWeele TJ, Arterburn D. Using the E-Value to Assess the Potential Effect of Unmeasured Confounding in Observational Studies. *JAMA*. 2019;321(6):602-603. doi:10.1001/jama.2018.21554

6. VanderWeele TJ, Ding P. Sensitivity Analysis in Observational Research: Introducing the E-Value. *Ann Intern Med*. 2017;167(4):268-274. doi:10.7326/M16-2607

© 2023 Inoue K et al. *JAMA Network Open.*
