# Supplementary material for: Association of Daily Step Patterns With Mortality in US Adults
Source: JAMA Netw Open. 2023 Mar 28;6(3):e235174. doi: 10.1001/jamanetworkopen.2023.5174 (PMC10051082; doi:10.1001/jamanetworkopen.2023.5174)
Supplement: Supplement 2. — Data Sharing Statement [file jamanetwopen-e235174-s002.pdf]

Data Sharing Statement

Inoue. Association of Daily Step Patterns With Mortality in US Adults. *JAMA Netw Open*. Published March 28, 2023. doi:10.1001/jamanetworkopen.2023.5174

Data
Data available: Yes
Data types: Deidentified participant data
How to access data: https://www.cdc.gov/nchs/nhanes/index.htm
When available: With publication

Supporting Documents
Document types: None

Additional Information
Who can access the data: anyone requesting the data
Types of analyses: for any purpose
Mechanisms of data availability: without investigator support
Any additional restrictions: The data is publicly available
